# Data-Enhancement Strategies in Weather-Related Health Studies

**DOI:** 10.3390/ijerph19020906

**Published:** 2022-01-14

**Authors:** Pierre Masselot, Fateh Chebana, Taha B. M. J. Ouarda, Diane Bélanger, Pierre Gosselin

**Affiliations:** 1 Department of Public Health, Environments and Society, London School of Hygiene and Tropical Medicine (LSHTM), 15–17 Tavistock Place, London WC1H 9SH, UK; 2 Institut National de la Recherche Scientifique, INRS, Centre Eau Terre Environnement, 490 rue de la Couronne, Québec, QC G1K 9A9, Canada; fateh.chebana@inrs.ca (F.C.); taha.ouarda@inrs.ca (T.B.M.J.O.); diane_belanger@bell.net (D.B.); pierre.gosselin@inspq.qc.ca (P.G.); 3 Institut National de Santé Publique du Québec, INSPQ, 945 av Wolfe, Québec, QC G1V 5B3, Canada; 4 Ouranos, Montréal, QC H3A 1B9, Canada

**Keywords:** environment, epidemiology, time series, aggregation, empirical mode decomposition (EMD), functional regression, weather, health, Canada

## Abstract

Although the relationship between weather and health is widely studied, there are still gaps in this knowledge. The present paper proposes data transformation as a way to address these gaps and discusses four different strategies designed to study particular aspects of a weather–health relationship, including (i) temporally aggregating the series, (ii) decomposing the different time scales of the data by empirical model decomposition, (iii) disaggregating the exposure series by considering the whole daily temperature curve as a single function, and (iv) considering the whole year of data as a single, continuous function. These four strategies allow studying non-conventional aspects of the mortality-temperature relationship by retrieving non-dominant time scale from data and allow to study the impact of the time of occurrence of particular event. A real-world case study of temperature-related cardiovascular mortality in the city of Montreal, Canada illustrates that these strategies can shed new lights on the relationship and outlines their strengths and weaknesses. A cross-validation comparison shows that the flexibility of functional regression used in strategies (iii) and (iv) allows a good fit of temperature-related mortality. These strategies can help understanding more accurately climate-related health.

## 1. Introduction

During recent years, the relationship between weather and human health has abundantly been studied. The harmful effect of heat waves [1,2,3] and cold spells [4,5] are now well documented. Other weather hazards, such as humidity [6,7], floods [8], and snowfalls or freezing rain [9], are now being analysed as well. There are several factors that affect weather-related health studies, such as air pollutants [10], aeolian activities [11], and pollen [12]. In a climate-change context, it is important to accurately represent the relationship between weather and health in order to be able to predict its evolution [13].

Evidence is still lacking in several areas of weather-related health. As an example, while extreme heat-related mortality and its projected increase seem to be widely accepted [14], the evolution of winter-related mortality is less clear [5,15]. Another example is the question of the impact of humidity and its role as a confounder in weather-related health studies, which is still open [16]. Some studies focusing on short-term effect found limited evidence that humidity plays a role [17], while others considering longer cumulative effects report an impact, especially on influenza [7,18]. Quantifying physiological adaptation for forecasting purpose is also an important challenge [19]. Uncovering these areas, as well as others, may be crucial for an efficient anticipation of climate change impacts on population health [20].

The most flexible and popular design to study climate-related impacts is probably time series [21]. However, time-series studies often rely solely on daily time series and restrict themselves to estimating day-to-day effects of the exposures of interest, which might explain part of the difficulty of obtaining conclusive results on several aspect of climate-related health [22]. The design flexibility offers nonetheless many options to study other temporal scales than daily, such as the cumulative effect over time, the evolution of the risk, as well as sub-daily effects [23].

The objective of this paper is to discuss four strategies to include additional temporal considerations to models. These strategies all rely on time-series data preprocessing to extract the features of interest, as summarized in Table 1. The first strategy is to temporally aggregate time series as a mean to control for short time confounders and estimate cumulative effects of the exposure (AG strategy for aggregation). The second focuses on exposure time series and seeks to simultaneously consider several time scales embedded in the data by first decomposing them through empirical model decomposition (EMDR strategy for empirical mode decomposition regression). The third and fourth strategy consider data as functional, i.e., as continuous curves in order to consider differential impacts of the climate exposure along the time domain of the curve. In the third, annual curves are considered to study the risk evolution across the year (FY for functional yearly), and in the fourth, daily curves of the exposure are considered for sub-daily risk estimation (FD for functional daily). We briefly introduce and discuss each strategy then apply and compare them on a real-world case study of temperature-related cardiovascular mortality risks in the metropolitan community of Montreal (MCM), Canada.

## 2. Materials and Methods

### 2.1. Data

Throughout the paper, the discussed strategies are illustrated on a dataset from the MCM, Canada. The dataset consists in daily cardiovascular mortality counts and temperature measures, both spanning the period from the 1st of January 1981 to the 31st of December 2011 (N=11,322 days). Cardiovascular mortality counts are provided by the national institute of Public Health of Quebec (Institut national de santé publique du Québec), including deaths attributed to ischaemic heart diseases (I20–I25 in the tenth version of the international classification of diseases, ICD-10), heart failure (I50 in the ICD-10), cerebrovascular diseases, and transient cerebral ischaemic attacks (G45, H34.0, H34.1, I60, I61, I63, and I64 in the ICD-10). Corresponding ICD-9 codes are selected for data before year 2000.

For strategies one to three (AG, EMDR, and FY), the temperature series are provided by Environment Canada and correspond to the spatial mean of several weather stations scattered throughout the MCM territory. For the FD strategy, hourly temperature series are provided by the Ministry of environment and climate change of Quebec (Ministère de l’environnement et de la lute contre les changements climatiques). These series start in 2007 and cover 5 years until ($N_{4}=1826$) and are also spatial means of several stations within the MCM.

### 2.2. Proposed Strategies

#### 2.2.1. AG Strategy: Aggregating the Health Response

Short-term confounding, mainly by week-end effect, is a known phenomenon in environmental epidemiology [24]. These short-term patterns can mask the effect of the exposure in low-population areas in which the number of cases is low. It is usually controlled for by including a day-of-week term in regression models. However, including such a term assumes these patterns are roughly constant over time, thus ignoring underlying annual trends. Including more terms, such as interaction with the year or months, would result in many coefficients to estimate and thus unstable models.

The proposed strategy is to aggregate health outcome time series prior to their inclusion in epidemiological model (AG strategy), thus smoothing out the short-term confounders, accounting for changes in their magnitude. AG strategy can be described with the two following steps: (i) temporally aggregate the health time series and (ii) perform a regression analysis with the aggregated health series as the response of the model. Based on a comparison between a variety of options, Masselot et al. [25] recommend the use of kernel smoothing on future values only (i.e., the weighting is null on the “left” of the current value) for the first step. In the present application, the cardiovascular mortality series is aggregated using the Epanechnikov kernel [26] with a window size of 7 days. This smooths a significant amount of the short confounding while maintaining the main patterns, such as important mortality episodes.

The second step can be performed with any regression model, and the present study considers the distributed lag nonlinear models (DLNM) [27]. The DLNM is fitted as in the international study of Gasparrini et al. [28], i.e., through quadratic b-splines with knots placed at the 10th, 75th, and 90th quantiles in the temperature dimension and with knots placed linearly on the logarithmic scale for the lag dimension. The maximum lag considered is 21 days. In addition, the long-term trend is controlled by a smooth spline with one degree of freedom per decade [29], and the seasonality is controlled through 4 sine/cosine pairs [21].

Since the aggregation of the first step creates an artificial autocorrelation in the response, the model additionally includes a time-series model (e.g., an autoregressive integrated moving average model) on the residuals of the regression model. An autoregressive model of order 5 (AR(5)) is considered, chosen by minimizing the Akaike information criterion (AIC) through a stepwise algorithm [30].

#### 2.2.2. EMDR Strategy: Empirical Mode Decomposition Regression

Weather and health are complex phenomena varying according to a very large number of factors. This complexity is apparent in the time-series data of weather variables and health issues, as they embed variations at different time scales. The purpose of the EMDR strategy is to retrieve the different time scales embedded in a time series to estimate which are the relevant ones for estimating weather/health relationships. The time scales are extracted through the EMD algorithm, [31] which decomposes a time series in a small number of basic oscillating components called intrinsic mode functions (IMF). Each IMF represents a particular frequency band existing in the series and, unlike Fourier series for instance, can be irregular to catch the variations in amplitude of natural variations.

To account for mode mixing, i.e., specific frequencies that are not represented during the whole series [32], the multivariate EMD (MEMD) is applied with additional white noise variables as described elsewhere [33]. White noise variables are then discarded from the final decomposition. This extension allows each IMF to represent a narrow frequency band, aiding both interpretation and the subsequent regression model. In this study, two white noise variables are added with a standard deviation equal to 20% of the standard deviation of temperature time series, as recommended in previous studies [34].

Yang et al. [35] showed that using IMFs as covariates in regression analysis instead of raw time series allowed to detect new patterns in weather/health relationships. Later, Qin et al. [36] and Masselot et al. [34] used the Lasso regression [37] to only keep the IMFs having a significant effect on the response in the model. The EMD-regression strategy then contains two steps: (i) decomposing weather variables into sets of IMFs and (ii) using these IMFs as the covariates of a Lasso regression to find the variations with the best predictive power of the health issue. More specifically, the regularization path algorithm is applied with a Poisson response [38] with the regularization parameter chosen by minimizing 10-fold cross-validation.

#### 2.2.3. FY Strategy: Annual Variations through Functional Regression

It has been shown that the risk of temperature varies within the year with, for instance, higher risks associated to heat early summer compared to late summer [39]. Similarly, Lee et al. [40] suggested that the relationship between mortality and temperature is not constant throughout the year, with a larger impact of cold during December than during January and February in the United States. These studies relied either on complex models with multiple interactions between variables for the former or on subdividing the data into months and fitting one model for each month, with both strategies resulting in loss of power.

This strategy (and the next one) proposes to consider time-series data as functional data, i.e., as a collection of continuous curves instead of a series of scalar values [41]. This provides a framework to model time-dependent processes, such as temperature and mortality [42]. More specifically, the FY strategy considers annual curves, i.e., each year of data is considered as the evaluation on a set number of times of a continuous curve. The underlying model is therefore a collection of 31 curves for both cardiovascular mortality and temperature.

The functional framework allows the application of a functional historical linear model that models each point of the outcome curve using a specific lag range from this point on the exposure curve [43]. This allows the lag-response curve to change smoothly across the year. The functional historical linear model is fitted through the general framework proposed by Brockhaus et al. [44]. This framework is fitted by a gradient boosting algorithm, allowing such complex models to be fitted by iteratively fitting simplified versions of the model (called base-learner) on the previous step residuals.

Specifically, this study considers a lag of 30 days with a base learner of cubic penalized splines with 4 degrees of freedom on both the day-of-year and lag dimensions. As the FY strategy is more suited to study the seasonal evolution, the model controls for the inter-annual trend through a smooth B-spline component with 3 degrees of freedom representing roughly one per decade. The boosting algorithm is fitted with a small step size of 0.1, and the optimal number of steps is chosen through 10-fold cross-validation, up to a maximum of 100 steps [45].

#### 2.2.4. FD Strategy: Intraday Variation through Functional Regression

It is usually difficult to incorporate information from the exposure at a smaller timescale than the outcome, such as using hourly temperature to assess the risk on daily mortality. One of the main issues is the collinearity created by using variables representing exposure separated by only one hour. However, by considering continuous curves instead of scalar variables, functional data analysis provides a framework for this kind of study [42,46].

This strategy is also based on functional data analysis, as this time considers hourly exposure values as daily curves of temperature. These functional observations are then fueled into a functional predictor regression [47] that estimates the impact of the temperature at each hour of the day on the daily death count. Similarly to the FY strategy, the functional predictor model is fitted through the general functional linear array model (FLAM) framework [48], estimated by gradient boosting. As the base learner is simpler than in the FY strategy, it is here chosen as a penalized spline with 2 regularly placed knots. A smooth time variable is also added to the model to account for the inter-annual mortality trend and a day-of-week factor. The boosting algorithm fitting the model is parametrized as in the FY strategy.

#### 2.2.5. Numerical Comparison

The four strategies are compared to a classical application of the DLNM, fitted as described in Section 2.2.1. The comparison is performed through the prediction error estimated using cross-validation (CV) in order to control for potential overfitting. In particular, this study considers a hv-block CV [49], i.e., the dataset is split by year of data, considering each year as the validation sample iteratively. The relative root mean squared error (rRMSE) curves are then computed, i.e., the RMSE is computed for each day of the year and is divided by this day’s average death count. This provides temporal information on the strengths and weaknesses of each strategy. Note that for the FD strategy, this information is computed only on the summer months as this model is designed for very short-term effects. Finally, once a daily rRMSE is obtained, the curves are further smoothed by locally weighted regression (LOESS) [50] for daily variation removal and better comparison between the models.

## 3. Results

**Figure 1:**
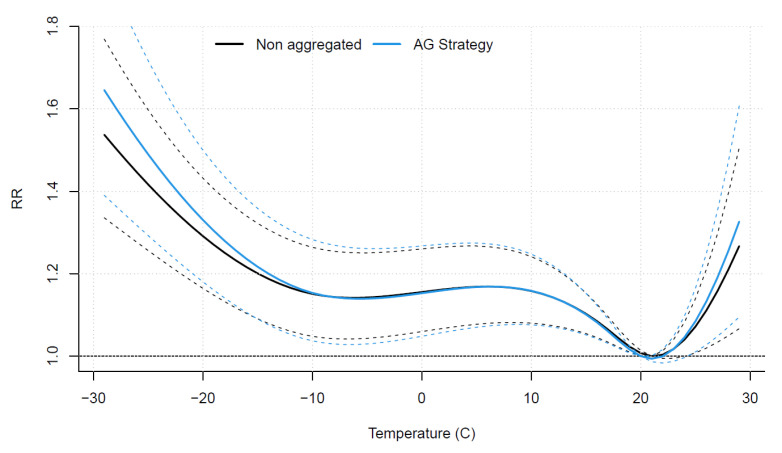
Estimated overall cumulative relative risk (RR) of temperature for the classical model and the first strategy (AG strategy). Dashed lines represent 95% confidence intervals.

The AG strategy smooths the cardiovascular mortality while preserving major events, such as the over-mortality of July 2010 in Montreal [51], as illustrated in Supplementary Materials (Figure S1). Figure 1 shows the overall cumulative relative risk (RR) of temperature for the AG strategy and the RR obtained with a DLNM fitted without aggregation of the series. The estimated relationships by the two models are similar, but the AG strategy shows slightly higher risks at both ends of the temperature range. Indeed, the mortality outcome due to these extremes might be scattered over several days, and aggregating the response allows better representing the overall impact of these extremes, especially cold.

**Figure 2:**
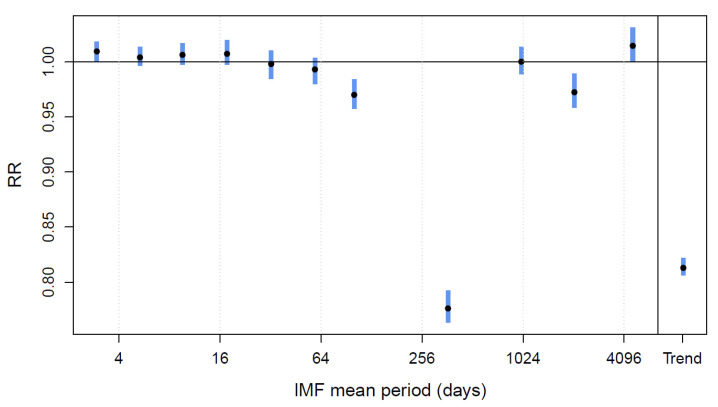
Relative risks (RR) of cardiovascular mortality associated to the temperature intrinsic mode functions (IMF) kept by the Lasso versus to the mean period of the IMF. Blue bars indicate 95% confidence intervals.

Applying the EMD on the temperature series results in 12 IMFs and an increasing residual trend that represent a warming of 1.4 °C over the 30 years of data. Figure 2 shows the RR associated to the amplitude of IMFs kept in the model by the Lasso. RRs significantly below one are associated to the IMF of periodicity of roughly 100 days that shows higher amplitude during winter and to the seasonality showing that mortality is usually lower in summer being around 80% the average of winter, confirming the overall impact of cold is more important than heat. A low RR is also associated to the trend representing the overall diminution of susceptibility across the years. A low RR is associated to the IMF with periodicity around 2000 days (roughly 5.5 years), which might correspond to the oscillation of low-frequency climate indices, such as the Atlantic multidecadal oscillation [52], although longer times series would be needed to be more conclusive. Finally, RRs above one are associated with the highest frequency IMFs, that have important amplitude especially during winter. During this season, such IMFs could represent important variations of temperature resulting in freezing rain or snowfalls, which are important stressors in the province of Quebec.

**Figure 3:**
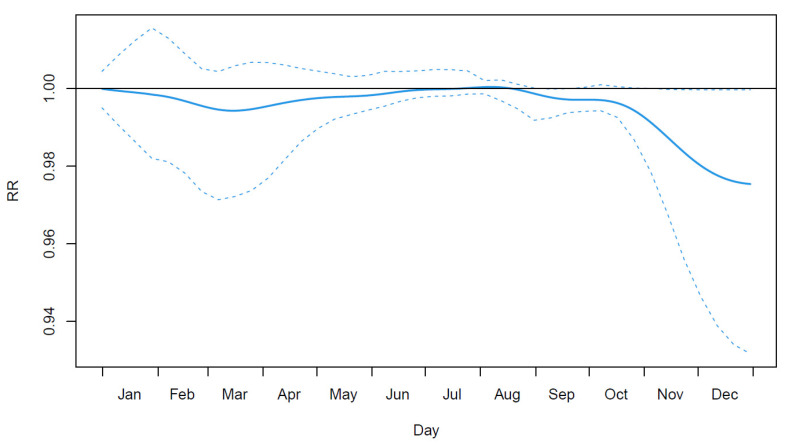
Estimated overall relationship between the cardiovascular mortality and temperature across the year. Dashed lines indicate 95% confidence intervals. This overall relationship is obtained by summing the functional coefficient along the lag dimension. Note that the seemingly low values of the relative risk (RR) are explained by its continuous nature (the relationship is spread across the whole curve).

Figure 3 shows the cumulative overall relative risk between cardiovascular mortality and temperature at each day of the year. Cold dominates the curve as it is mostly negative, especially early and late winter, i.e., transitional periods during which cold is more unusual. This result is consistent with the findings of Lee et al. [40] in the United States but does not necessitate the fitting of a large number of models; neither does it require the arbitrary separation of months. In addition, the curve is slightly above one during summer, as heat is the main exposure during this period.

**Figure 4:**
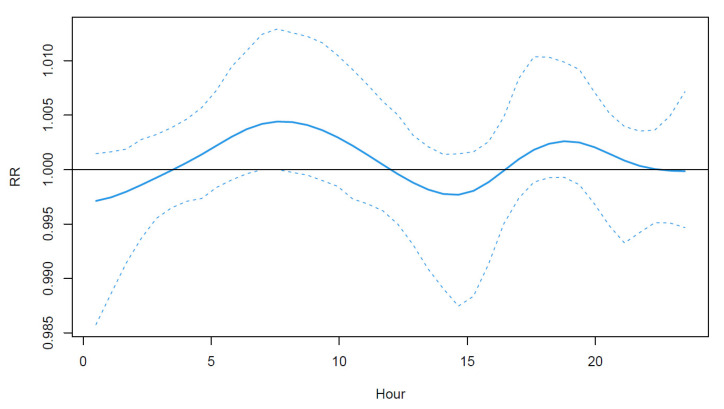
Estimated relationship between cardiovascular mortality count and the previous day temperature. From left to right, hours correspond to midnight of previous day to midnight of current day. Dashed lines indicate 95% confidence intervals. Seemingly low values of relative risk (RR) are due to the spreading of the risk along the whole day.

Figure 4 shows the strategy FD results, i.e., the estimated RR associated to the previous day temperature during summer. The functional is the highest during morning and evening, i.e., during periods that are not usually the hottest of the day. This roughly corresponds to hours during which people are usually commuting, and thus, a larger proportion of the population is exposed to heat since the air conditioning prevalence is relatively high in Montreal [53]. Although it is known that the minimum temperature plays a role in heat-related mortality since it is often included in heat-health warning systems [54,55], the functional model clarifies this aspect of heat-related mortality.

**Figure 5:**
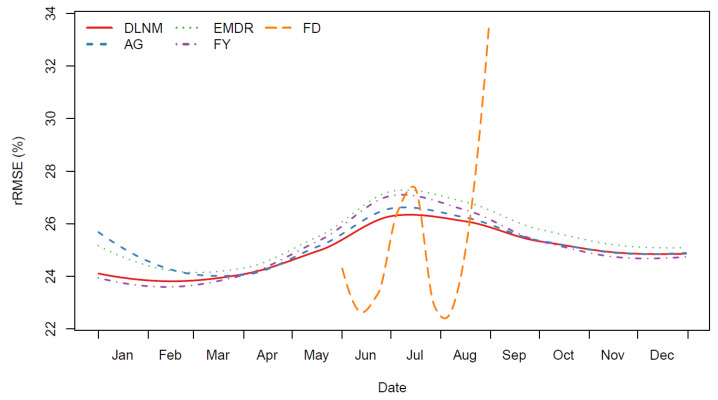
Cross-validated relative RMSE (rRMSE) along the year for each strategy and the benchmark model. The rRMSE is defined as the square root of the mean square prediction error divided by the mean of the raw response. In this figure, the computed rRMSE is smoothed by locally weighted regression (LOESS). DLNM, distributed lag nonlinear model; AG, aggregation of response; EMDR, EMD-regression; FD, functional with daily curves; FY, functional at the yearly level.

Figure 5 shows the smoothed rRMSE across the year for each strategy. The AG, EMDR, and FY strategies show curves with similar patterns being better during winter than summer while also outlining strengths. The AG curve is overall the highest of all, while the FY and DLNM are the lowest. The EMDR curve shows as good performances as the DLNM during early winter and spring, periods during which short-term variations of temperature might have higher importance. The FY strategy is the best during winter overall, as it focuses more on middle-term impacts during the year. Finally, the FD curve is overall lower during summer, especially in June and August, suggesting this hourly information is important for these periods that are not at the heart of summer.

## 4. Discussion

In the context of weather-related health studies, the present paper argues that time-series data contains information that can be exploited by some preprocessing of the series. Several strategies are discussed to extract this information from data according to the objective of the study and the characteristics of available data. This extraction can be made either by removing irrelevant information (AG strategy), discriminating information at different scales (EMDR strategy), or even by changing the nature of data from scalar to functional (FY and FD strategies). Although the present paper does not dive deep into the details of each strategy, appropriate references are provided for the detailed application of these strategies.

This study applies the proposed strategies to estimate temperature-related cardiovascular mortality in the census area of Montreal, Canada. As this is a cold city, most strategies outline the importance of cold more than heat in the impact of temperature (except for the FD strategy more suited to heat). It especially shows that the impact of cold is spread compared to heat and that the risk is higher early in winter as well as during spring. The latter period can still see cold spells happening although the weather is warming. Some important variations of temperature during winter can also be the source of over-mortality. The FD strategy focusing more on heat potentially shows the protective effect of air conditioning, as the risk is higher at periods during which the population tend to commute. The comparison between these strategies also shows the relative strength of each of them, with FY being especially useful in winter, while FD is useful in summer.

**Figure 6:**
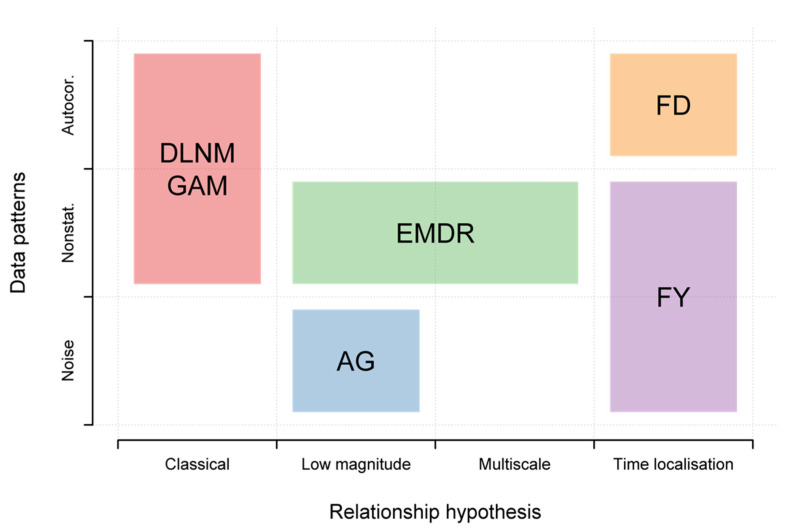
Summary of the cases of interest for each strategy. The abscissa indicates the scale corresponding to the objective of the study and the ordinate to the issues potentially present in the data. DLNM, distributed lag nonlinear model; GAM, generalized additive model; AG, aggregation of response; EMDR, EMD-regression; FD, functional with daily curves; FY, functional at the yearly level.

All of the assumptions made on data leading to the strategies outlines on the present paper are linked to statistical patterns present in the data. Indeed, when a relationship of low magnitude is assumed for the AG strategy, this means that high frequencies of series are considered to be noise for the purpose of the study. Hence, AG strategy is intended to be used when data are thought to be noisy and that this noise could hinder the study. EMDR strategy is meaningful since all data series considered in environmental epidemiology are nonstationary. Instead of controlling for dominant patterns causing nonstationarity, the EMDR strategy integrates them to the analysis. Nonstationarity is also a key argument in favor of the FY strategy because, since data are whole years, the seasonality is automatically controlled by the strategy. FD strategy could have been conducted with 24 explanatory variables, but this would have resulted in strong collinearity issues because of the autocorrelation of the series. Therefore, FD strategy is addressing autocorrelation issues in data series [56] with the assumption that successive measurement are one and only continuous datum. Figure 6 summarizes the relationship assumptions and underlying statistical patterns addressed by each strategy.

A main limitation of the strategies discussed here is that they are all linear with the exception of the AG strategy. The results obtained with these strategies are detailed, complex, and already show important potential with performances on the same level as the DLNM, which is the current state-of-the-art in time series studies. Masselot et al. [34] showed that the complex nonlinear weather-health relationship can be decomposed in simpler linear ones. However, the linearity of the method still limits their application as, for instance, high frequencies of the EMDR strategy could integrate both heat and cold effect that a linear coefficient cannot represent accurately. Similarly, both the FY and FD strategies could be significantly improved with a nonlinear association at each point of the curve. However, considering nonlinear models would greatly increase the complexity of these strategies and their interpretation and thus represent non-trivial methodological development. Although nonlinear functional models have been proposed in the past [57], their application is still limited with, for instance, no historical effect integrated.

Another limitation of the proposed strategies is the necessity of an important amount of data for them to reach high performances. Although the EMDR strategy can be useful for studying a relationship at the interannual scale, the concerned IMF needs to have several cycles completed for the estimated relationship to be accurate. For the FY strategy, the application uses only 31 years of data, i.e., only n=31 curves, which is hardly enough for estimating such a complex surface as the one in Figure 3. However, this limitation will lose its relevance in the future with the acquisition of new data.

## 5. Conclusions

The time-series design is a flexible way to assess the impact of weather on health outcomes. Four strategies are proposed to enhance the information in time series to study the relationship at various time scales and periods. These strategies include aggregating the health outcome, decomposing the weather time series into its different variation modes, and considering annual and daily functional data. It is recommended to carefully consider the objective of the study and use the most adapted strategy. Typically, the EMDR and AG strategy are well suited to long-term studies, while both functional strategies can accommodate well time-varying effects. Nevertheless, applying all strategies to temperature-related cardiovascular mortality in Montreal (Canada) provides information on the impact of both heat and cold. These strategies may prove useful for environmental epidemiology and can contribute to more efficient action planning in future climate.

## Figures and Tables

**Table 1:** Summary of each strategy including its objectives and illustrations. In each strategy, prior modifications of data are shown in blue, and data used directly are shown in grey.

			Illustration	
Strategy	Description	Objectives	Health Response	Weather Exposure
AG	Aggregated response	- Diminish noise influence in the health response - Estimate longer-term variations in the health response	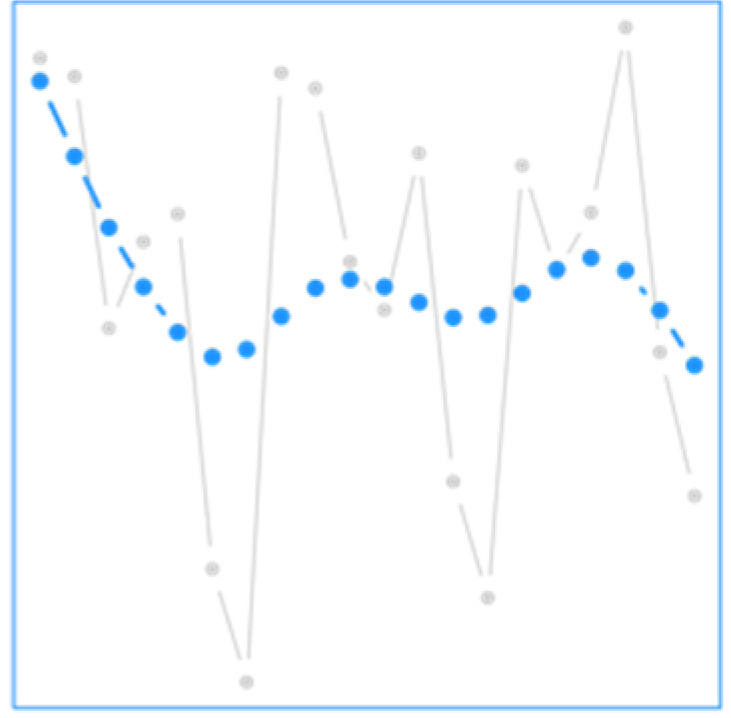	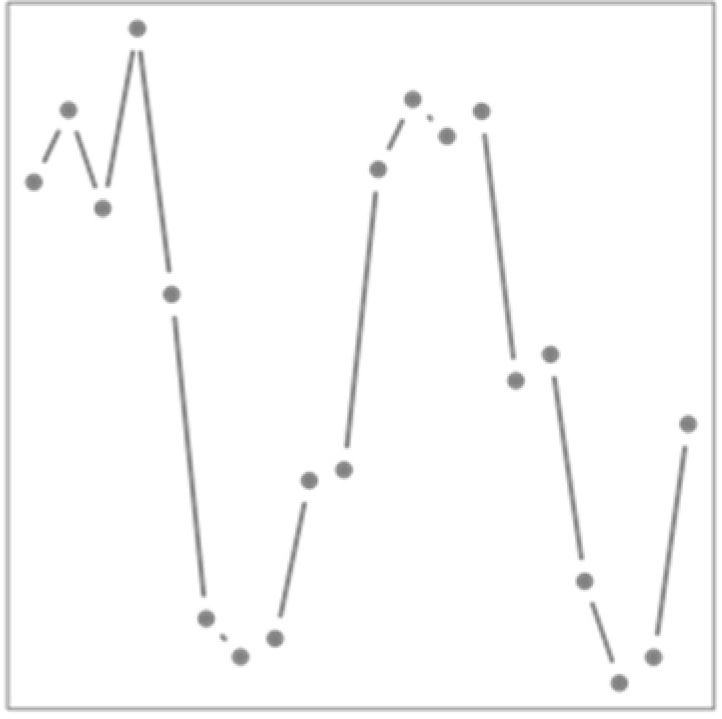
EMDR	EMD-regression	- Study classical and non-dominant time scales separately	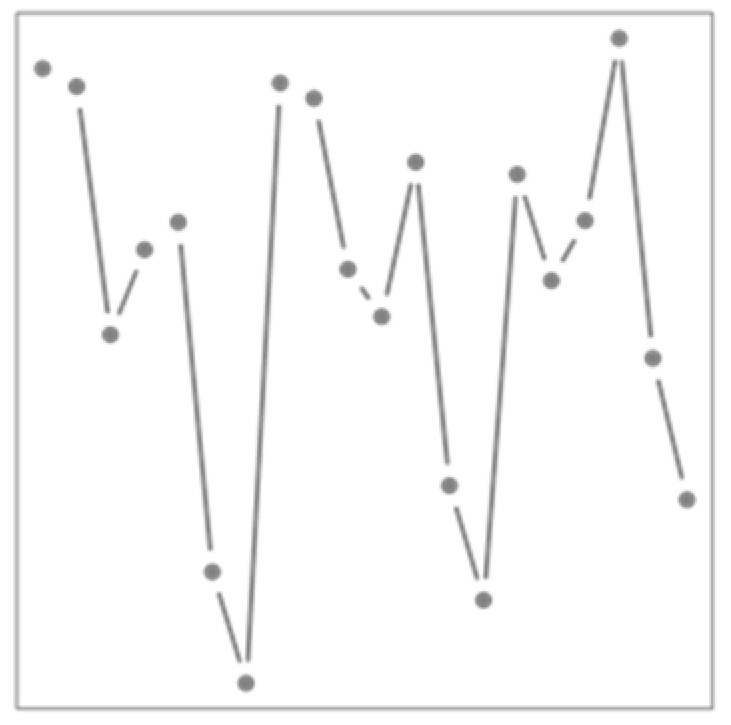	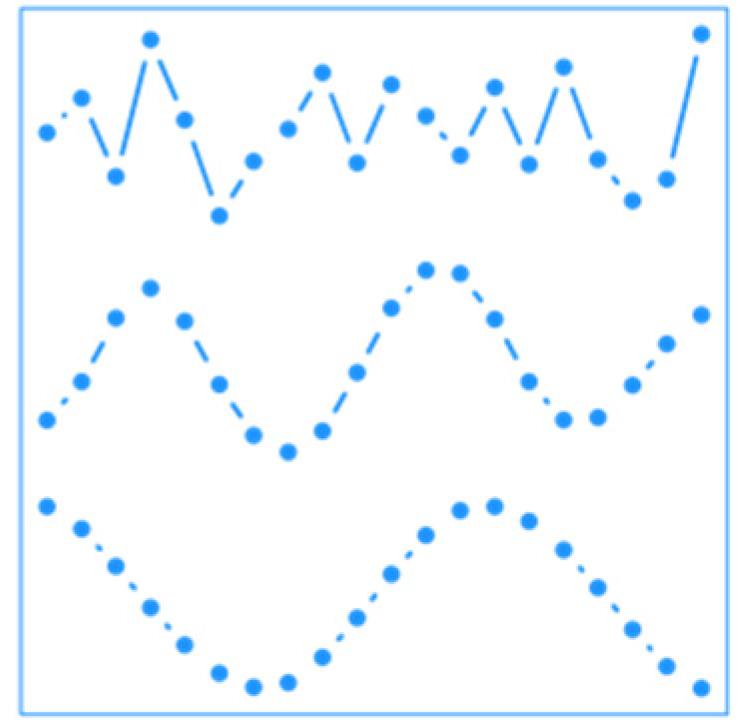
FY	Functional regression at the yearly level	- Estimate the evolution of weather/health relationship across the year	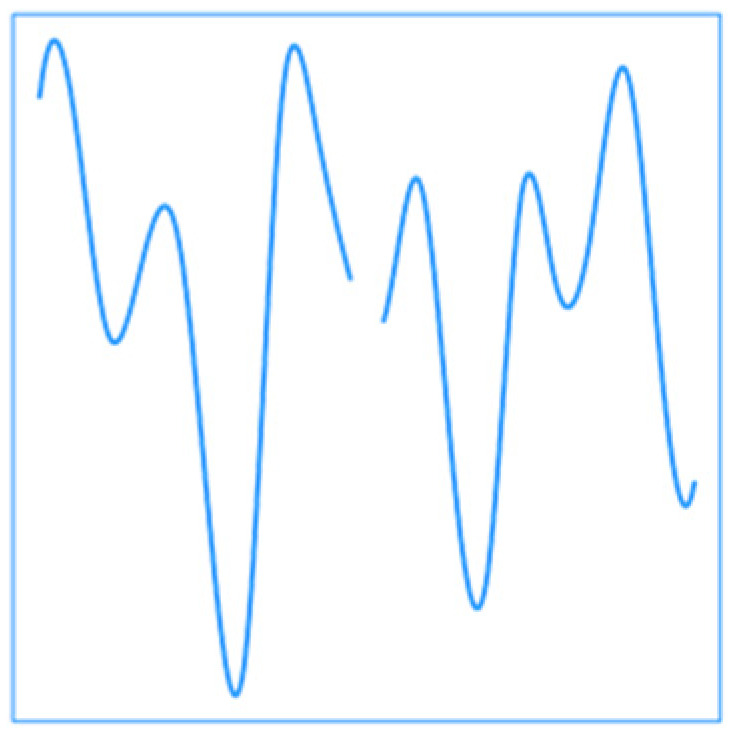	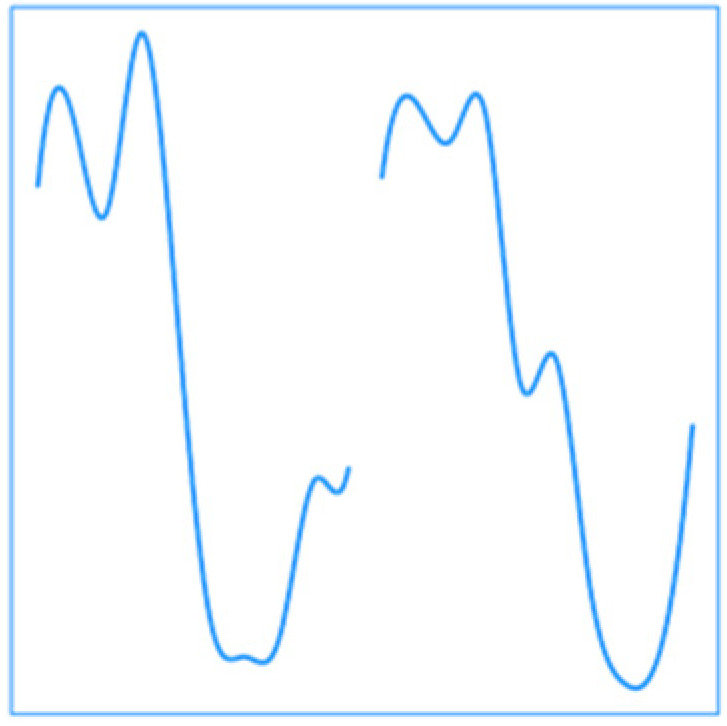
FD	Functional regression at the daily level	- Estimate the effect of intraday weather variations on health issues	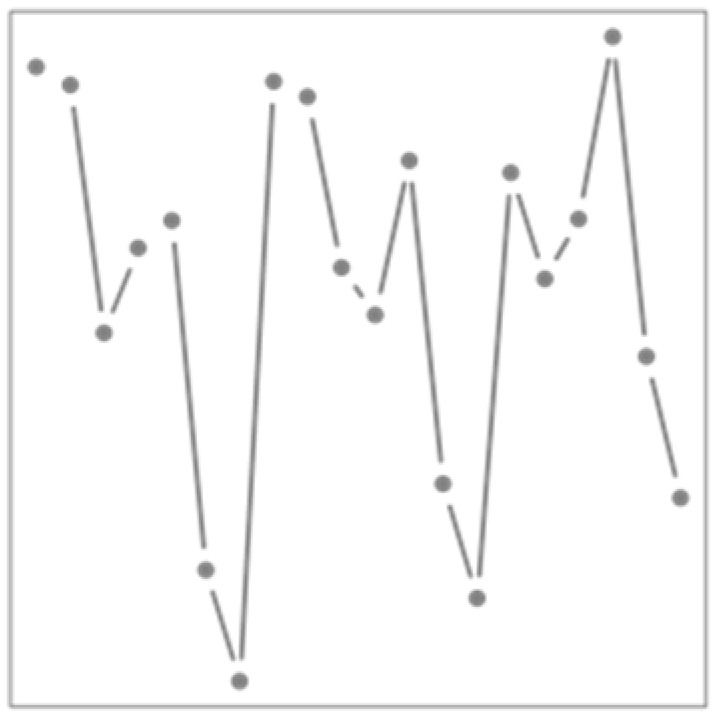	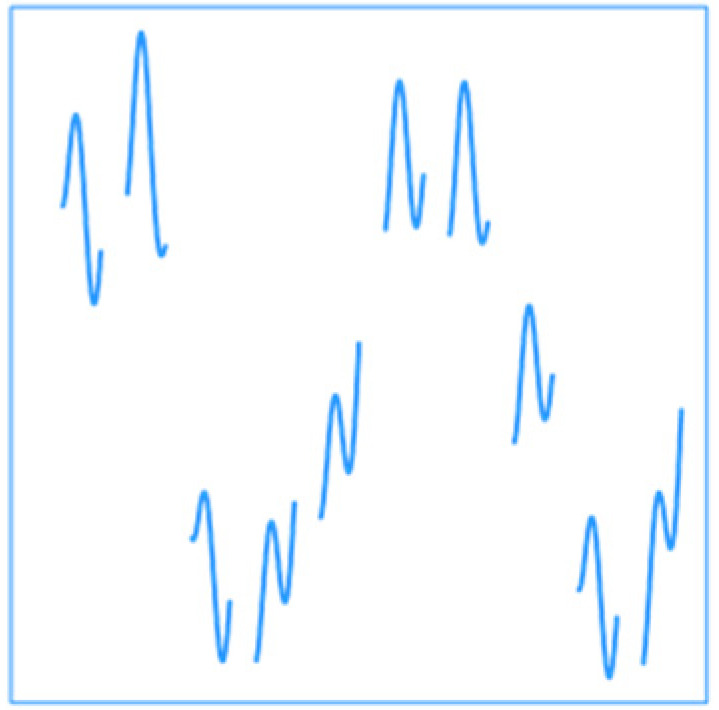

## Data Availability

Data are not available, due to governmental privacy policy.

## Supplementary Materials

The following are available online at https://www.mdpi.com/article/10.3390/ijerph19020906/s1, Figure S1: Aggregated mortality of year 2010 used in AG strategy; Figure S2: Decomposed temperature series used in EMDR strategy.
