# Supplementary material for: Data-Enhancement Strategies in Weather-Related Health Studies
Source: Int J Environ Res Public Health. 2022 Jan 14;19(2):906. doi: 10.3390/ijerph19020906 (PMC8776088; doi:10.3390/ijerph19020906)
Supplement: Supplementary file 1 [file ijerph-19-00906-s001.zip › ijerph-1500291-supplementary.pdf]

Supplementary information

Data enhancement strategies in weather-related health studies

Pierre Masselot[1*], Fateh Chebana[1], Taha B.M.J. Ouarda[1],

Diane Bélanger[1,2], Pierre Gosselin[1,2,3]

[1]Institut National de la Recherche Scientifique, Centre Eau-Terre-Environnement, Québec, Canada;

[2]Centre Hospitalier Universitaire de Québec, Centre de Recherche, Québec, Canada;

[3]Institut national de santé publique du Québec (INSPQ), Québec, Canada.

**Corresponding Author: pierre-lucas.masselot@ete.inrs.ca*

Figure S1 shows, as an example, the aggregated mortality of year 2010 for Strategy 1. Aggregation is performed through Nadaraya-Watson kernel smoothing with a 7-day Epanechnikov kernel. Note that the window includes only future values, *i.e.* the aggregation at time t is computed with observations at times $t, \ldots, t+6$. The aggregated series shows that Strategy 1 smooth a non-negligible amount of noise from mortality data. However, notable events are kept in the series, such as the important over-mortality event during the July heat wave (see Bustinza et al., 2013).

Figure S2 shows the IMF obtained by decomposing the temperature series through EMD. More precisely, here the noise-assisted EMD of Rehman et al. (2013) is used here, with two noise variables having a standard deviation equal to 10% of the temperature's standard deviation. Note that consecutive IMF spanning similar frequencies have been summed to yield the IMF $C8$ and $C11$ of Figure S2. Figure S2 shows that the temperature seasonality and trend due to climate change are well represented in the IMF.

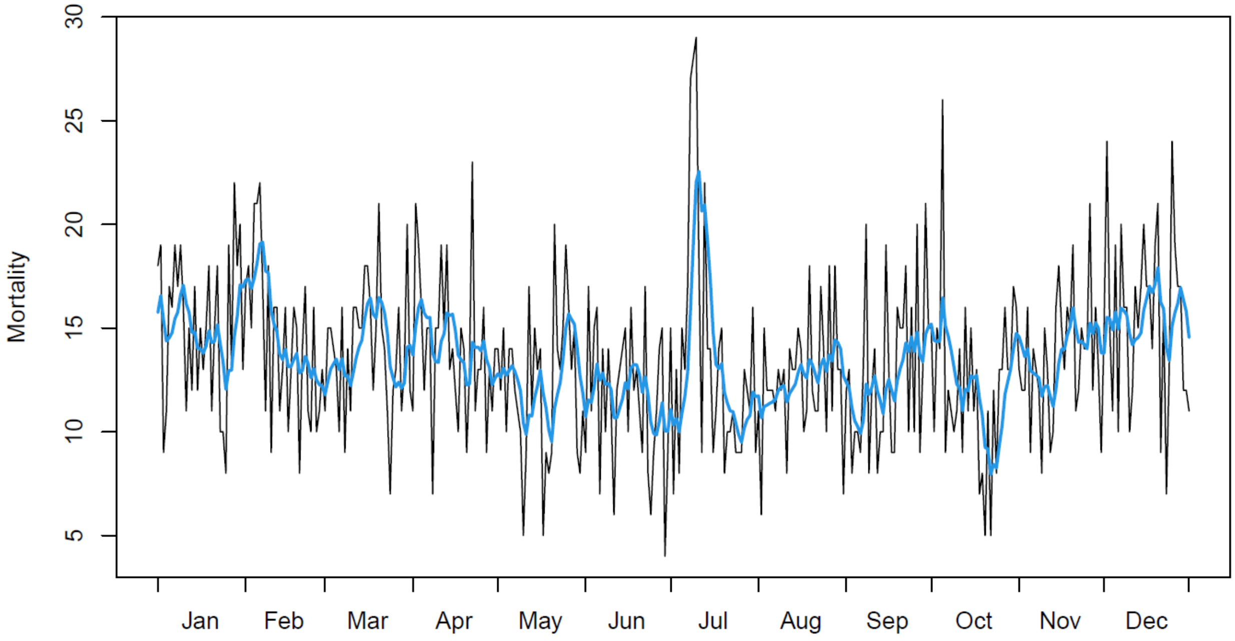

Figure S1. Aggregated mortality of year 2010 used in AG strategy.

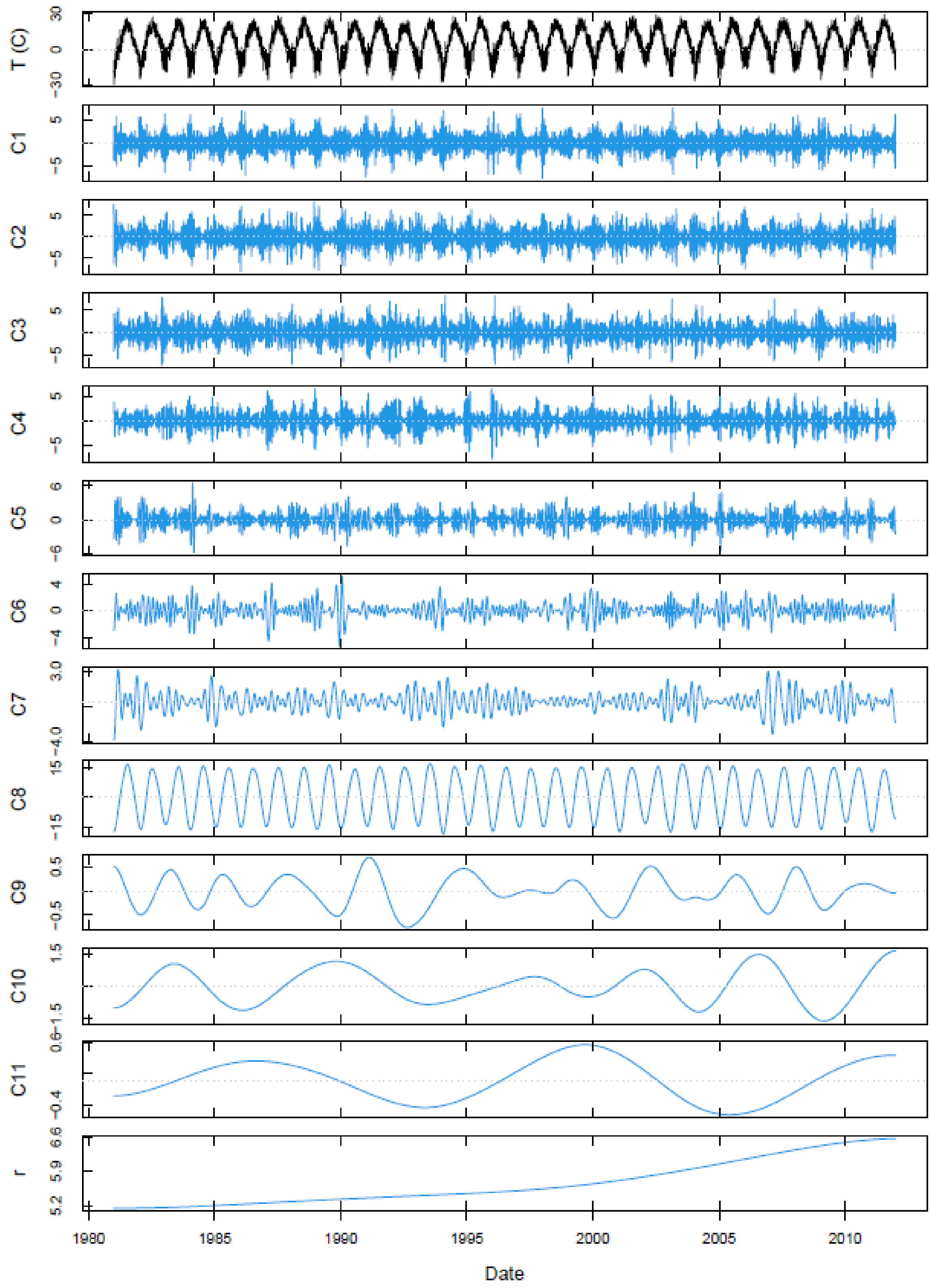

Figure S2. Decomposed temperature series used in EMDR strategy.

References

Bustinza, R., Lebel, G., Gosselin, P., Belanger, D., Chebana, F., 2013. Health impacts of the July 2010 heat wave in Quebec, Canada. BMC Public Health 13, 56.

Rehman, N.U., Park, C., Huang, N.E., Mandic, D.P., 2013. EMD Via MEMD: Multivariate Noise-Aided Computation of Standard EMD. Adv. Adapt. Data Anal. 05, 1350007. https://doi.org/10.1142/S1793536913500076
